# Calretinin immunohistochemical staining in Hirschsprung’s disease: An institutional experience

**DOI:** 10.14744/nci.2020.69376

**Published:** 2021-12-31

**Authors:** Ebru Zemheri, Pinar Engin Zerk, Cigdem Ulukaya Durakbasa

**Affiliations:** 1. Department of Pathology, University of Health Sciences, Umraniye Training and Research Hospital, Istanbul, Turkey; 2. Department of Pathology, University of Health Sciences, Okmeydani Training and Research Hospital, Istanbul, Turkey; 3. Department of Pediatric Surgery, Medeniyet University Goztepe Training and Research Hospital, Istanbul, Turkey

**Keywords:** Calretinin, hirschsprung, immunohistochemical staining

## Abstract

**Objective:**

This study aims to evaluate the results obtained by calretinin staining on tissue samples for diagnosing Hirschsprung’s disease (HD) in a single institution, by single expert.

**Methods:**

A retrospective evaluation was done for calretinin immunostaining in HD patients for a period of 3 years. Calretinin staining was evaluated in nerve fibers. Calretinin immunohistochemistry was considered positive if any staining was seen in nerve fibers and/or ganglion cells in the lamina propria, muscularis mucosa or submucosa. According to staining intensity, staining was classified as strong, weak or negative. The pathological diagnosis was based on presence or absence of ganglion cells (G0/G1) and nerve hypertrophy (N0/N1). Samples were classified according to the depth (presence of submucosa or intermuscular area), the type (biopsy or resection specimen) and staining intensity of calretinin (strong, weak, or negative staining).

**Results:**

A total of 96 tissue samples from 56 patients were studied. Tissues were from colon (43.8%), rectum (43.8%), stoma (6.2%), ileum (3.1%) and appendix (3.1%). The pathological diagnosis was G0N0 in 14.6%, G1N0 in 54.2%, G0N1 in 25% and G1N1 in 6.2% of cases. Our materials consisted of 92 tissue biopsies and four resection specimens. Intermuscular layer was present in 87.5% of materials and 12.5% of biopsies contained submucosa. Calretinin staining was negative (C0) in 37.5% of cases, strong positive (C1) in 47.9%, and weak positive (C2) in 14.6%. When the C0 category was taken as the reference, the status of calretinin staining as C2 (weak positive) in cases with pathological diagnosis of G1N0 was found to be 37.575 times that of cases with G0N0 (OR [95% CI]: 37.575 [2.928, 482.176], p=0.006) and the status of calretinin staining as C1 (strong positive) in cases with pathologic diagnosis of G1N0 was found to be 131.401 times that of G0N0 (OR [95% CI]: 131.401 [9.263, 1864.082), p<0.001).

**Conclusion:**

Calretinin staining is positive whenever ganglion cells are present independent from presence of nerve hypertrophy, the depth and the site of the biopsy or staining intensity. It is negative in all aganglionic samples. Calretinin staining is a reliable ancillary test in HD diagnosis.

**H**irschsprung’s disease (HD) is among the most commonly observed underlying pathologies for neonatal intestinal obstruction with a prevalence of one in 5000 births [1–3]. There is an intrinsic abnormality of the enteric sensory system in which ganglion cells of both submucosal (Meissner) and myenteric (Auerbach) nerve plexuses are absent. The definitive diagnosis of HD is done histopathologically by recognizing the absence of ganglion cells in the rectum through the affected length of bowel. Although there are suggestive clinical and radiological findings, the diagnostic gold standard is rectal biopsy. Demonstrating the absence of ganglion cells could be troublesome and repetitive hematoxylin-eosin (H&E) sections and ancillary technics such as acetylcholinesterase (AChE) and/or calretinin staining may be needed [1]. Although AChE staining has been improved to be the gold standard for the histopathological diagnosis of HD, it has not gained worldwide acceptance because its evaluation is technically difficult and problematic [4].

Calretinin is a calcium-binding protein which assumes a paramount role in the development and functioning of central nervous system. Over the past years, several studies evaluated the use of calretinin immunostaining as a diagnostic tool in HD and reported favorable results. While calretinin immunostaining is seen in the nerve fibers of muscularis mucosa and lamina propria in normal tissues, the staining is lost in HD [5–9].

The present study reports the experience of a single institution over 3 years comparing the status of calretinin immunostaining of ganglionic and aganglionic segments in full-thickness rectal biopsies as well as resection specimens in HD.

## Materials and Methods

This is a retrospective study including the HD patients of a single institution over 3 years (2013–2016). All specimens were assessed by a single pediatric pathologist (EZ) who has an experience in evaluation of tissue samples in HD.

Calretinin (monoclonal mouse antihuman antibody [DAKO]), (CLONE: DAK-Calret 1, Code: IR627) immunohistochemical staining was performed to all chosen paraffin-embedded blocks after routine H&E evaluation. Immunohistochemical staining was performed with a Bond Autostainer. Biopsies were localized as appendix, colon, ileum, rectum, and stoma according to biopsy site. The materials were classified as “biopsy” or “resection material” according to the surgical procedure.

Pathological diagnosis was based on absence or presence of ganglion cells (G) and nerve hypertrophy (N).

G0N0: No ganglion cells, no nerve hypertrophy

G1N0: Ganglion cells are present, no nerve hypertrophy

**Highlight key points**

• Diagnosis of HD can be challenging especially for pathologists who do not confront often with this disease, in biopsies taken superficially or in neonates who may have immature ganglion cells.
• Calretinin staining results, independent of biopsy site, depth of biopsy, or type of material whether it’s a biopsy or resection.
• Calretinin staining is an important and reliable diagnostic tool that complements the H&E examination.

G0N1: No ganglion cells, nerve hypertrophy present

G1N1: Ganglion cells are present, nerve hypertrophy present

The resection materials and the other biopsies were classified into groups according to the presence of submucosa and intermuscular area.

**Figure 1.**
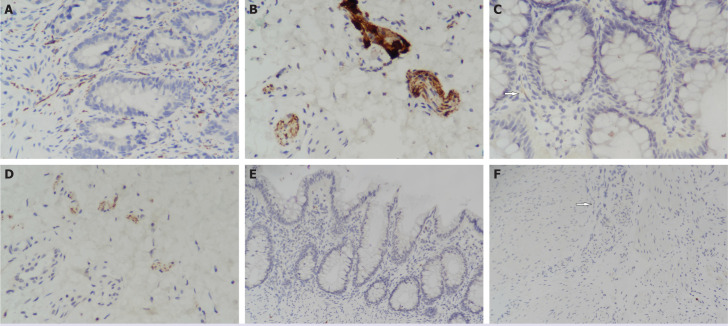
**(A)** Lamina propria shows linear nerve fibrils with a granular pattern of strong reactivity on calretinin IHC (calretinin IHC ×400: non-HD case). **(B)** Strong expression of calretinin in the nerve trunks (calretinin IHC ×400: non-HD case). **(C)** Lamina propria shows scattered linear nerve fibrils (arrow) weak reactivity on calretinin IHC (calretinin IHC ×400: non-HD case). **(D)** Weak expression of calretinin in the nerve trunks (calretinin IHC ×400: non-HD case). **(E)** Negative staining in lamina propria (calretinin IHC ×200: HD case). **(F)** Negative staining in nerve trunks (arrow) (calretinin IHC ×200: HD case).

Calretinin staining was evaluated in nerve fibers seen in lamina propria, submucosa and intermuscular area. Calretinin immunohistochemistry was considered positive if any staining in nerve fibers and/or ganglion cells was seen in the lamina propria, muscularis mucosa, or submucosa. According to staining intensity, the staining was classified as strong, weak or negative (Fig. 1).

### Statistical Analysis

Number Cruncher Statistical System 2007 (Kaysville, Utah, USA) program was used for statistical analysis. Descriptive statistical methods (mean, standard deviation, frequency, percentage, minimum, and maximum) were used when data were evaluated. Generalized linear mixed models (generalized linear mixed models) were used to investigate the effects of localization, pathological diagnosis, depth, and type of material on calretinin staining. In all models, while the dependent variable was calretinin staining, the other variables were taken as independently and a separate model was created for each variable. P<0.05 was accepted as statistically significant.

## Results

There were 56 patients. The mean age of patients ranged from 3 days to 13 years. There were 43 (76.8%) males and 13 (23.2%) females. As multiple biopsies are taken from the same patient on the same or different occasions, the total number of tissue samples were 96 with 92 biopsies and four resection materials. The tissue samples were from colon in 42, rectum in 42, stoma in six, ileum in three, and appendix in three. The intermuscular area was present in 84 of samples and 12 contained submucosa. The pathological diagnosis was G0N0 in 14 samples, G1N0 in 52, G0N1 in 24, and G1N1 in 6. Calretinin staining intensity was negative in 36 samples, weakly positive in 14, and strongly positive in 46 (Table 1).

**Table 1.** The distribution of tissue samples according to biopsy site, pathological diagnosis, depth of biopsy, type of material, and calretinin staining status

	n
Biopsy site	
Colon	42
Rectum	42
Stoma	6
Ileum	3
Appendix	3
Pathological diagnosis	
G0N0	14
G1N0	52
G0N1	24
G1N1	6
Depth of biopsy	
Submucosa	12
Intermuscular area	84
Type of material	
Biopsy	92
Resection	4
Calretinin staining status	
C0 Negative	36
C1 Strongly positive	46
C2 Weakly positive	14

Generalized linear mixed model evaluation showed that the localization, the depth or the type of the biopsy were not effective on results obtained by Calretinin staining (p=0.931, p=0.327, and p=0.999, respectively). The pathological diagnosis was significantly correlated with the calretinin staining results (F: 5.407, p<0.001) (Table 2).

**Table 2.** The effect of the localization, the pathological diagnosis, the depth of biopsy, and the type of material on calretinin staining

	F	p
Localization	0.376	0.931
Pathological diagnosis	5.407	<0.001*
Depth	1.133	0.327
Type of material	0.000	0.999

Generalized linear mixed models (F: Variant analysis, p: p value). *: P<0.05.

When the negative C0 category was taken as a reference, the status of calretinin staining as C2 (weakly positive) in cases with pathological diagnosis of G1N0 was found to be 37.575 times that of cases with G0N0 (OR [95% CI]: 37.575 [2.928, 482.176], p=0.006). With the same reference, the status of calretinin staining as C1 (strongly positive) in cases with pathologic diagnosis of G1N0 was found to be 131.401 times that of G0N0 (OR [95% CI]: 131.401 [9.263, 1864.082), p<0.001] (Table 3).

**Table 3.** Relation of staining intensity and pathological diagnosis

	Term	B	p	Exp (B)	95% CI
					Lower
C2 weakly positive	Intercept	–2.424	0.032		
	G0N0	–	–	–	–
	G1N0	3.626	0.006*	37.575	2.928
	G0N1	0.088	0.948	1.092	0.077
	G1N1	17.646	0.994	4.61*10^7^	0.000
C1 strongly positive	Intercept	–2.344	0.052		
	G0N0	–	–	–	–
	G1N0	4.878	<0.001*	131.401	9.263
	G0N1	–0.958	0.546	0.384	0.017
	G1N1	19.342	0.993	2.51*10^8^	0.000

C0 negative category for calretinin staining and G0N0 category for pathological diagnosis were taken as reference categories. *: P<0.05 (p: p value, B: Bias, Exp (b): Regression analysis).

## Discussion

HD is a complex congenital disease with a pathogenesis which has not been completely understood. Its molecular diagnosis has shown notable advance with growing evidence regarding involvement of specific genes in the development of enteric nervous system and migration of ganglion cells [10]. Mutations in RET gene on chromosome 10q11.2 was shown to be responsible for approximately 40% of sporadic HD cases. HD can also be seen as a familial disorder or associated with other syndromes or chromosomal abnormalities [11–14]. In these cases, multiple genes are thought to be involved in the pathogenesis. These molecular and genetic alterations may possibly be used in diagnosis of HD in upcoming years.

HD is one of the major causes of congenital intestinal obstruction. It’s characterized by the absence of ganglion cells both in submucosal and myenteric nerve plexuses. Due to the aganglionosis in the intestinal segment, the cholinergic activity is increased [15]. It is diagnosed by combination of clinical, radiological, and histopathological findings. A comparison of several diagnostic techniques showed that rectal biopsy was superior to the others with a sensitivity of 93% and a specificity of 100% [16].

The classical histopathological diagnosis of HD depends on examination of formalin-fixed paraffin-embedded tissues. Diagnosis of HD is characterized by the absence of ganglion cells on mucosa and muscularis mucosa with H&E staining [17]. It can be challenging especially for pathologists who do not confront often with this disease or under some specific circumstances. Difficulty occurs especially in biopsies taken superficially or in neonates who may have immature ganglion cells. Both may yield false-positive results, especially if a pathologist has not vast experience on such biopsies [18]. Some ancillary methods such as histochemical AChE staining may be used to avoid the false-positive results. In one study, the sensitivity and specificity of AChE histochemistry was found to be 93.5% and 100.0%, respectively [19]. AChE histochemistry definitely has some advantages but it has technical difficulties such as the need for fresh frozen tissue and complex technical equipment as well as interobserver and intraobserver discordance [19–23]. Because of such limitations of AChE staining, new markers were searched by investigators to facilitate the diagnosis of HD. Among several immunochemical markers, calretinin was discovered to identify ganglion cells, by Barshack in 2004 [5].

Calretinin, a Vitamin D-dependent calcium-binding protein, is a sensitive marker for ganglion cells and nerve fibers [1–3, 5]. It is a protein involved in calcium transport and modulates neuronal excitability. Absence of this protein leads to accumulation of calcium in cytoplasm. Excess calcium in the cytoplasm increases neuroexcitability and eventually leads degeneration in nerve fibers [23, 24]. Calretinin immunostaining is helpful in challenging cases with intestinal obstruction and more specifically, in diagnosing HD. It is prudent that calretinin immunohistochemistry should always be utilized as a part of H&E examination because the disease has a heterogeneous nature. Calretinin immunostaining is readily applied to paraffin-embedded specimens and the evaluation is done either as “positive” or “negative.” Therefore, it is an ideal tool in those cases with a challenging diagnosis. Ganglion cells and nerve trunks of the submucosa and subserosa are stained with calretinin in normal rectal and intestinal biopsies. In addition, nerve fibrils of the superficial submucosa, muscularis mucosae, and lamina propria also show immunoreactivity with calretinin in a linear and granular pattern. On the other hand, there is no immunostaining in nerve fibrils of the lamina propria, muscularis mucosa, or superficial submucosa in aganglionic intestinal segments of HD [1, 2, 5, 6].

Several studies compared calretinin staining to AchE staining. One study postulated evaluation of histopathology and AChE staining had difficulties and calretinin staining can be used safely without false- positive staining [25]. Zuikova et al. [26] proposed that calretinin is more useful and assessment of calretinin is easier than AChE staining. Jeong et al. [19] in 2008 showed in his study that although AChE histochemistry is a useful method in diagnosis of HD, usage of calretinin and AChE simultaneously will increase the chance of correct diagnosis of HD. Kapur et al. [21] reported that although calretinin staining has no misdiagnoses between observers, AChE staining has significantly more interobserver disagreement.

Various research studies comparing the staining status of calretinin in HD and in control group have been reported before. In these reports, it was found that nerve fibers in lamina propria, submucosa, and muscularis mucosa being positive in both control group and ganglionic segment of HD whereas it was negative in aganglionic segments of HD cases. The sensitivity and specificity ratios of calretinin staining in HD were variable; 93.3–100% and 100–93.8%, respectively [2, 9, 27]. H&E examination is paramount for diagnosis of HD and calretinin staining can be helpful in diagnosis, but staining should be evaluated carefully, because of possible false-positive results [3, 4, 28]. Despite false-positive results, calretinin staining is a useful marker in diagnosis, especially in cases with clinical suspicion of HD, in cases with immature ganglion cells or in which ganglion cells are few in number [6, 17, 29–31]. It should be taken into consideration that negative calretinin staining in the mucosa supports the diagnosis of HD but does not exclude aganglionosis in biopsies taken from distal rectum [30].

Our study shows that calretinin staining results, independent of biopsy site, depth of biopsy, or type of material whether it’s a biopsy or resection, are reliable. We, therefore, postulate even superficial biopsies that contain mucosa and submucosa only will be sufficient for calretinin staining to diagnose of HD. When ganglion cells are present in H&E examination, calretinin staining was positive independent from nerve hypertrophy. Calretinin staining was negative in cases which did not contain any ganglion cells. This finding was consistent with the previously published data stating H&E examination which is the gold standard and also calretinin staining is a reliable ancillary test in diagnosis of HD. It is, therefore, valuable especially in problematic cases. The presence of ganglion cells may result in weak or strong staining with calretinin so this is not a discriminating finding in diagnosis. Although strong calretinin staining was more common in presence of ganglion cells, any calretinin staining should be taken as a sign of ganglionic intestine independent from the intensity of the staining.

### Conclusion

The gold standard histopathological diagnosis of HD can be challenging because of several factors such as the biopsy depth, the patient age or the experience of the pathologist. H&E examination serial and multiple sections may be needed for the presence of ganglion cells. Calretinin staining is an important and reliable diagnostic tool that complements the H&E examination.
